# Risk of sudden sensorineural hearing loss in stroke patients: A 5-year nationwide investigation of 44,460 patients

**DOI:** 10.1097/MD.0000000000004841

**Published:** 2016-09-09

**Authors:** Chin-Lung Kuo, An-Suey Shiao, Shuu-Jiun Wang, Wei-Pin Chang, Yung-Yang Lin

**Affiliations:** a Institute of Brain Science, National Yang-Ming University; b Department of Neurology, Neurological Institute, Taipei Veterans General Hospital; c Laboratory of Neurophysiology; d Integrated Brain Research Laboratory, Taipei Veterans General Hospital; e Faculty of Medicine, National Yang-Ming University School of Medicine; f Department of Otolaryngology-Head and Neck Surgery, Taipei Veterans General Hospital; g School of Health Care Administration, Taipei Medical University; h Institute of Physiology, National Yang-Ming University; i Institute of Clinical Medicine, National Yang-Ming University, Taipei; j Department of Otolaryngology, Taoyuan Armed Forces General Hospital, Taoyuan; k Department of Otolaryngology-Head and Neck Surgery, Tri-Service General Hospital, National Defense Medical Center, Taipei, Taiwan, ROC.

**Keywords:** hearing loss, population, steroid therapy, stroke, sudden, Taiwan

## Abstract

Poststroke sudden sensorineural hearing loss (SSNHL) can hinder communication between patients and healthcare professionals, thereby restricting participation in rehabilitation programs and limiting improvements in physical performance. However, the relationship between stroke and SSNHL remains unclear. This study employed a nationwide population-based dataset to investigate the relationship between stroke and SSNHL.

The Taiwan Longitudinal Health Insurance Database was used to compile data from 11,115 stroke patients and a comparison cohort of 33,345 matched nonstroke enrollees. Each patient was followed for 5 years to identify new-onset SSNHL. Stratified Cox proportional-hazard regression analysis was used to examine the association of stroke with subsequent SSNHL.

Among the 44,460 patients, 66 patients (55,378 person-years) from the stroke cohort and 105 patients (166,586 person-years) from the comparison cohort were diagnosed with SSNHL. The incidence of SSNHL was approximately twice as high among stroke patients than among nonstroke patients (1.19 and 0.63/1000 person-years, respectively). Stroke patients had a 71% increased risk of SSNHL, compared with nonstroke patients (adjusted hazard ratio [HR] 1.71, 95% confidence interval [CI] 1.24–2.36). We also observed a remarkable increase in risk of SSNHL in stroke patients within 1-year of follow-up (adjusted HR 5.65, 95% CI 3.07–10.41) or under steroid therapy during hospitalization (adjusted HR 5.14, 95% CI 2.08–12.75).

Patients with stroke had a higher risk of subsequent SSNHL compared with patients without stroke. In particular, stroke patients within 1-year follow-up and those undergoing steroid therapy during hospitalization should be treated with the utmost caution, considering that the risk of SSNHL increases by more than 5-fold.

## 1 Introduction

Sudden sensorineural hearing loss (SSNHL) is an otologic emergency that occurs over a period of less than 72 hours.^[1]^ SSNHL can be a frightening experience for patients, particularly those who are critically reliant on hearing for work, such as musicians or professional drivers. SSNHL can have a tremendous impact on patient quality of life, and is associated with a high risk of adverse cognitive and functional outcomes. The etiology of SSNHL has only been identified in 10% of cases. Furthermore, in cases where etiology has been identified, stroke has been identified as one of the most common causes.^[2]^

Stroke is a leading driver of neurological disability and placement in long-term care. A third of stroke survivors are functionally dependent on others 1 year after stroke onset.^[3]^ A comprehensive rehabilitation program could help patients become more independent; however, SSNHL can hinder communication between patients and healthcare professionals, thereby restricting participation in rehabilitation programs and limiting improvements in physical performance.^[4]^ Previous large population-based research has also shown that hearing loss is associated with an increase in all-cause mortality through mediating variables, including walking disability, cognitive impairment, and self-rated health.^[5]^ Previous research which analyzed National Health Insurance (NHI) data revealed that SSNHL was associated with a significant increase in the risk of stroke during the subsequent 5 years of follow-up, suggesting that SSNHL may be an early indicator of stroke.^[6]^ Therefore, although a previous hospital-based study failed to reveal a sequential association between SSNHL and stroke,^[7]^ clinical evidence to indicate a connection between stroke and SSNHL nonetheless exists.

Given a potential link between stroke and SSNHL, it is reasonable to hypothesize an inter-relationship between the clinical profiles of stroke patients and poststroke SSNHL. The theoretical expectation, however, has yet to be fully elucidated, and a lack of relevant evidence precludes the formulation of clear recommendations with regard to clinical practice. Clearly, a deeper understanding of SSNHL risk in stroke patients is required; therefore, this study employed a large-scale population-based cohort to elucidate and confirm our hypothesis.

## 2 Methods

### 2.1 National Health Insurance Research database

This study was approved by the Taipei Veterans General Hospital's Institutional Review Board (VGHIRB NO. 2015-10-004CC). In 1995, the Taiwanese government implemented a compulsory NHI mechanism. The NHI oversees the reimbursement of healthcare costs for up to 99% of the 23.5 million residents of Taiwan.^[8]^ All claims data are collected in the NHI research database and managed by the Taiwan National Health Research Institute (NHRI). The NHI research database includes comprehensive medical data, including records of registration, ambulatory and inpatient care, catastrophic illness, and a variety of information related to drug prescriptions.^[6,8]^

All data used in this study were retrieved from the Longitudinal Health Insurance Database 2005 (LHID2005)—a subset of the NHI research database. The LHID2005 contains longitudinal data (1996–2010) on medical claims for 1,000,000 individuals randomly selected from the 2005 Registry of Beneficiaries (n = 23.72 million) of the Taiwan NHI program. The NHRI seeks to eliminate all statistically significant differences in age or sex between the randomly sampled group and beneficiaries of the NHI program. Hundreds of peer-reviewed studies have employed data from the Taiwan NHI, confirming the high validity of data from the NHI program.^[8–12]^

### 2.2 Study design and population

This research employed a study cohort and a comparison cohort to investigate the relationship between stroke and subsequent development of SSNHL. The study cohort consisted of patients who had been newly diagnosed with any type of stroke (international classification of diseases, ninth revision, clinical modification (ICD-9-CM) codes 430 to 438) between January 1, 1997 and December 31, 2005. For these patients, the date of initial stroke diagnosis was assigned as the index date. To enhance the precision of inclusion criteria related to stroke, we considered only the following: patients whose stroke had been diagnosed by neurologists, neurosurgeons, or internal physicians; and patients with ≥2 ambulatory visits or ≥1 inpatient visits for stroke. We excluded subjects aged less than 18 years. Assembly of a comparison cohort involved the random matching of 3 patients who had not suffered a stroke to a patient in the stroke cohort based on age, sex, and index year. Patients who had a pre-existing diagnosis for stroke or SSNHL were excluded from both cohorts.

### 2.3 Outcome variables

Outcome variables were based on the occurrence of SSNHL (ICD-9-CM code 388.2). To increase the diagnostic validity of SSNHL, inclusion criteria required that patients had received ≥2 ambulatory visits or ≥1 inpatient visit; and SSNHL diagnostic codes had been assigned by an otolaryngologist.

### 2.4 Adjusted co-variables

Potential confounders of the association between stroke and SSNHL, including hypertension, hyperlipidemia, and diabetes, were extracted from the claims data.^[6]^ These factors were included in regression models as co-variables. Previous research has revealed that the development of stroke is associated with various sociodemographic characteristics, including level of urbanization, monthly income, and geographic location of the community in which the patient resided.^[6,13]^ We also included these sociodemographic characteristics in regression models as adjusted co-variables.

In accordance with criteria established by the NHRI, towns and cities in Taiwan were stratified into 7 urbanization categories, with 1 indicating the highest level of urbanization and 7 indicating the lowest. Criteria included population density (persons/km^2^), percentage of people with a college-level education or higher, percentage of people aged 65 years or older, percentage of agricultural workers in the local population, and number of physicians per 100,000 people.^[6]^ The number of stroke cases were low in areas with an urbanization level of 4, 5, 6, and 7; therefore, these levels were combined into a single urbanization group referred to as level 4.

### 2.5 Statistical analysis

Pearson chi-square tests were used to examine differences in categorical data between stroke and comparison cohorts. Parametric continuous data related to the 2 cohorts, including follow-up duration, were compared using the Student *t* test.

We calculated the incidence rate of SSNHL by dividing the number of new cases by the number of person-years at risk for a given period. The number of person-years at risk was defined as the number of patients at risk times the number of years between respective measurements (i.e., from the entry dates to either: the censored dates of SSNHL occurrence, withdrawal from follow-up, loss to follow-up, death, or the end of the study period, whichever came first). The Kaplan–Meier method was used to calculate the SSNHL-free rate for all patients diagnosed with stroke between the date of the first hospitalization or ambulatory visit for stroke and the censored dates of SSNHL occurrence, withdrawal from follow-up, loss to follow-up, death, or the end of the study period (December 31, 2010), whichever came first. We applied the log-rank test to examine differences in SSNHL-free rates between the 2 cohorts. Cox proportional-hazard regression analysis was used to examine the risk of SSNHL in the stroke and comparison cohorts during the 5-year follow-up period. Adjustments were made for age, sex, urbanization level, monthly income, geographic region, hypertension, hyperlipidemia, and diabetes. Hazard ratios (HRs) and 95% confidence intervals (CIs) were calculated to represent the risk of SSNHL in each of the cohorts.

To assess whether the risk of SSNHL in stroke patients was affected by clinical factors, we determined the HRs for SSNHL in both cohorts, the results of which were stratified according to the observation period, and also age and sex. We sought to determine whether the risk of SSNHL in stroke patients was affected by the type of stroke. Based on the ICD-9-CM coding system, stroke patients (codes 430 to 438, n = 11,115) were categorized as follows: cases of hemorrhagic stroke (codes 430–432, n = 1176); cases of ischemic stroke (codes 433–435, n = 6419); and ill-defined cases of stroke (codes 436–438, n = 3520). To ensure the accuracy of data, ill-defined cases of stroke (n = 3520) were excluded from further HR analysis. Instead, we sought to determine the HRs for SSNHL in hemorrhagic and ischemic cohorts. We further used the Kaplan–Meier method to determine the 5-year SSNHL-free rates for nonstroke patients and patients with ischemic stroke and hemorrhagic stroke.

We also sought to determine whether steroids have preventative effects on the likelihood of stroke patients developing SSNHL. Cox proportional-hazard regression analysis was used to compare the HRs of SSNHL in stroke patients who underwent steroid therapy during hospitalization, patients who did not undergo steroid therapy during hospitalization, and the comparison cohort. Patients who underwent steroid therapy for any reason after discharge were excluded. All data analyses were conducted using SAS software (SAS Institute Inc., Cary, NC). A *P* value <0.05 was considered statistically significant.

## 3 Results

### 3.1 Demographic characteristics

A total of 44,460 patients were included in the study, including 11,115 patients in the stroke cohort and 33,345 patients in the comparison cohort. Statistically significant differences were observed in urbanization level and monthly income. Furthermore, stroke patients were more likely than nonstroke patients to have comorbidities such as hypertension, hyperlipidemia, and diabetes (all *P* < 0.001) (Table 1).

**Table 1:**
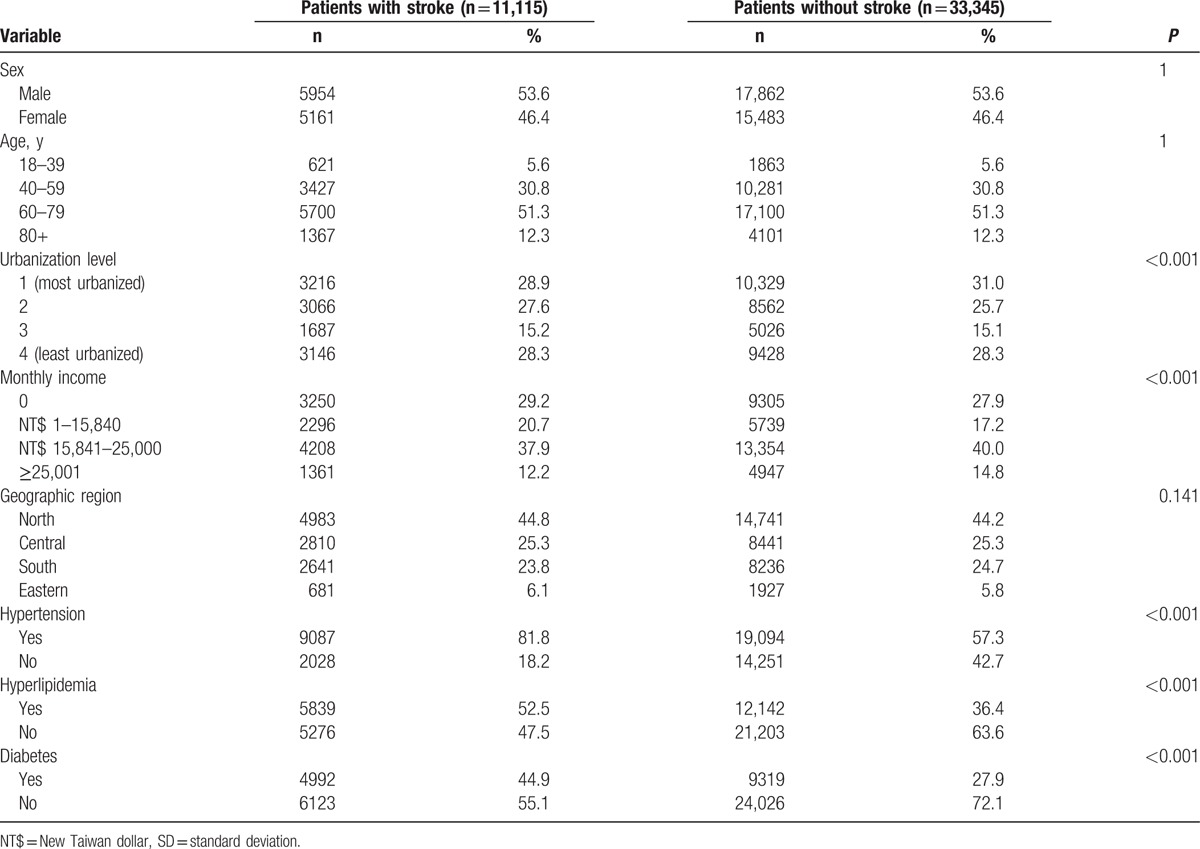
Demographic characteristics and comorbid disorders for patients with/without stroke (N = 44,460).

### 3.2 Incidence rates of SSNHL

During the 5-year follow-up, a total of 171 patients developed SSNHL, including 66 patients in the stroke group and 105 patients in the comparison group. The overall incidence density was approximately 2-fold higher in the case cohort (1.19 per 1000 patient-years) than in the comparison cohort (0.63 per 1000 patient-years). Furthermore, the higher incidence densities observed in stroke patients were irrespective of age, sex, and follow-up period. The highest incidence density of SSNHL was noted in stroke subjects 40 to 59 years of age (1.76 per 1000 patient-years) and in control subjects 60 to 79 years of age (0.74 per 1000 patient-years). Males had a higher incidence density of SSNHL than females, and this was true for both stroke patients (1.38 vs 0.97 per 1000 patient-years) and nonstroke patients (0.76 vs 0.48 per 1000 patient-years) (Table 2).

**Table 2:**
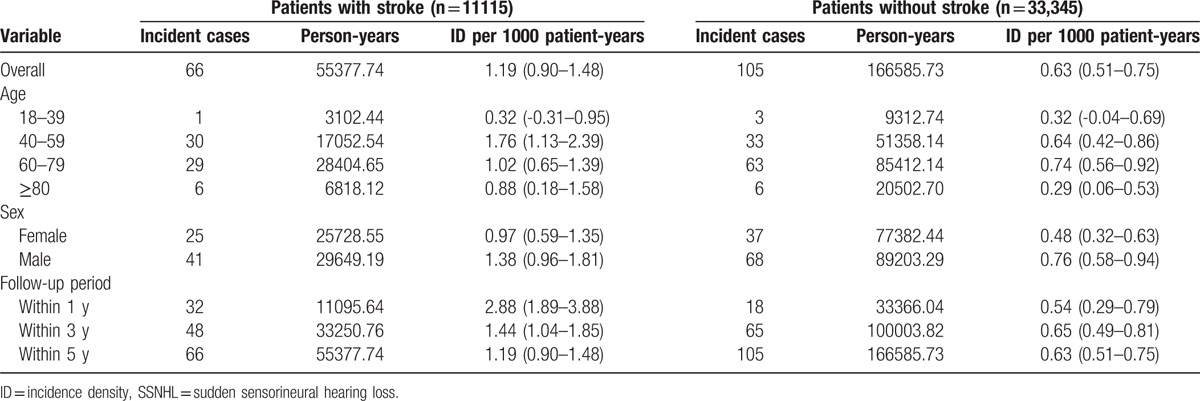
Overall, age, sex, and follow-up period-specific incidence densities of SSNHL for the 2 cohorts (n = 44,460).

It should be noted that the incidence density of SSNHL among patients with stroke gradually decreased over time, from 2.88 to 1.19 per 1000 patient-years. In contrast, the incidence density of SSNHL among patients without stroke remained relatively steady at 0.54 to 0.65 per 1000 patient-years throughout the follow-up period. These observations indicate that a more severe ischemic effect exists during the period close to the onset of stroke. These findings also underline the importance of using a stroke severity scale to assess the association between stroke and subsequent SSNHL. Unfortunately, the ICD-9-CM coding system does not provide specific codes by which to designate the severity of stroke. Further comparative longitudinal prospective studies will be required to clarify our observations.

### 3.3 Increased risk of SSNHL in stroke patients

**Figure 1:**
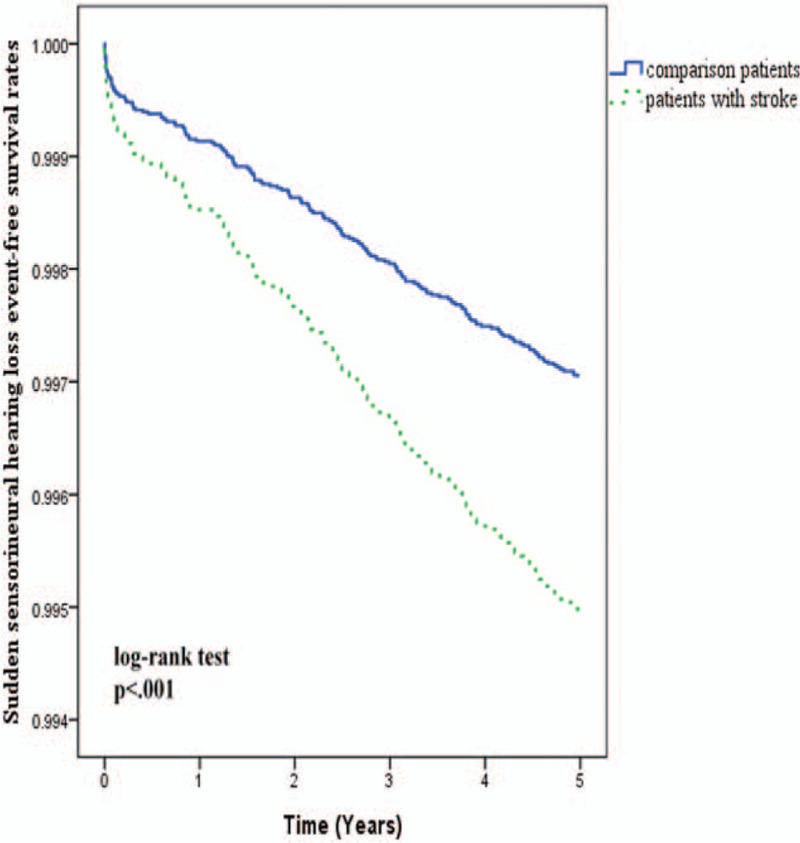
Sudden sensorineural hearing loss-free rates among patients with and without stroke during the 5-year follow-up period.

The results of Kaplan–Meier survival analysis (Fig. 1) revealed that the 5-year SSNHL-free rate in patients with stroke was significantly lower than in the comparison cohort (log-rank test, *P* < 0.001). In other words, the 5-year incidence of SSNHL in stroke patients was significantly higher than that of the control group. This finding is consistent with the results of Cox regression analysis, which indicated that the crude HR of SSNHL was 1.89 times higher among stroke patients (95% CI 1.39–2.57, *P* < 0.001) than among those in the comparison cohort (Table 3). After adjusting for potential confounders, stroke patients were 1.71 times more likely to develop SSNHL than patients without stroke (95% CI 1.24–2.36, *P* = 0.001).

**Table 3:**
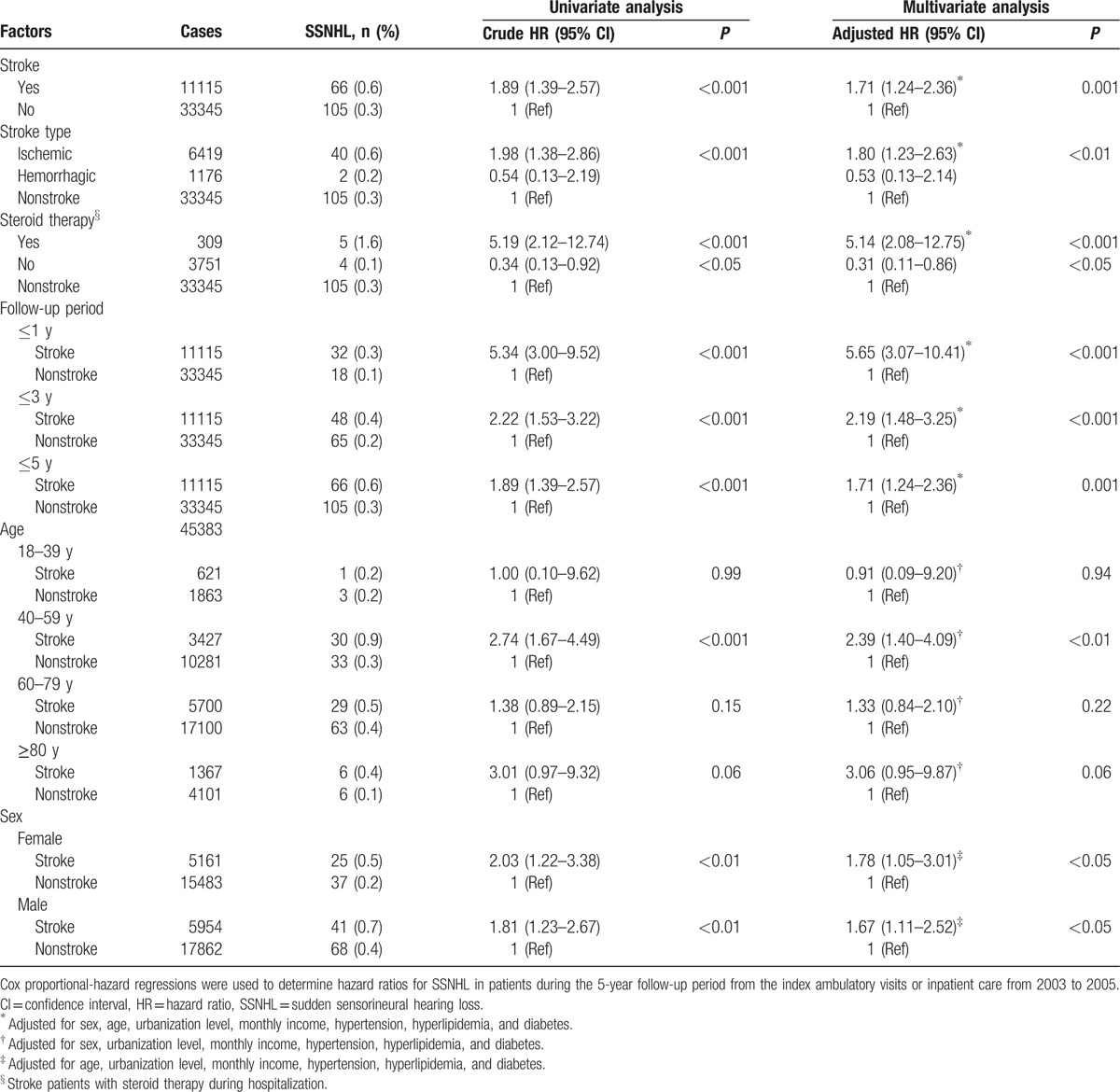
Hazard ratios for SSNHL in stroke and comparison patients stratified by clinical factors.

### 3.4 Impact of stroke subtypes on risk of SSNHL

Among the 1176 cases of hemorrhagic stroke, 2 patients subsequently developed SSNHL during the subsequent 5-year follow-up period. Among the 6419 cases of ischemic stroke, 40 patients developed SSNHL during the 5 years of follow-up. Among the 3520 ill-defined cases of stroke (excluded to ensure data accuracy), 24 patients developed SSNHL during the 5-year follow-up. In total, 66 patients developed SSNHL after stroke.

We assessed whether the risk of SSNHL in stroke patients was affected by the type of stroke (i.e., ischemic or hemorrhagic stroke). Compared with nonstroke patients, those with ischemic stroke presented a greater likelihood of developing SSNHL before (HR 1.98, 95% CI 1.38–2.86, *P* < 0.001) and after (HR 1.80, 95% CI 1.23–2.63, *P* < 0.01) adjusting for potential confounders (Table 3). It should be noted that the reverse situation was observed when patients with hemorrhagic stroke were compared with nonstroke patients (adjusted HR 0.53), although the difference was not significant (*P* > 0.05).

**Figure 2:**
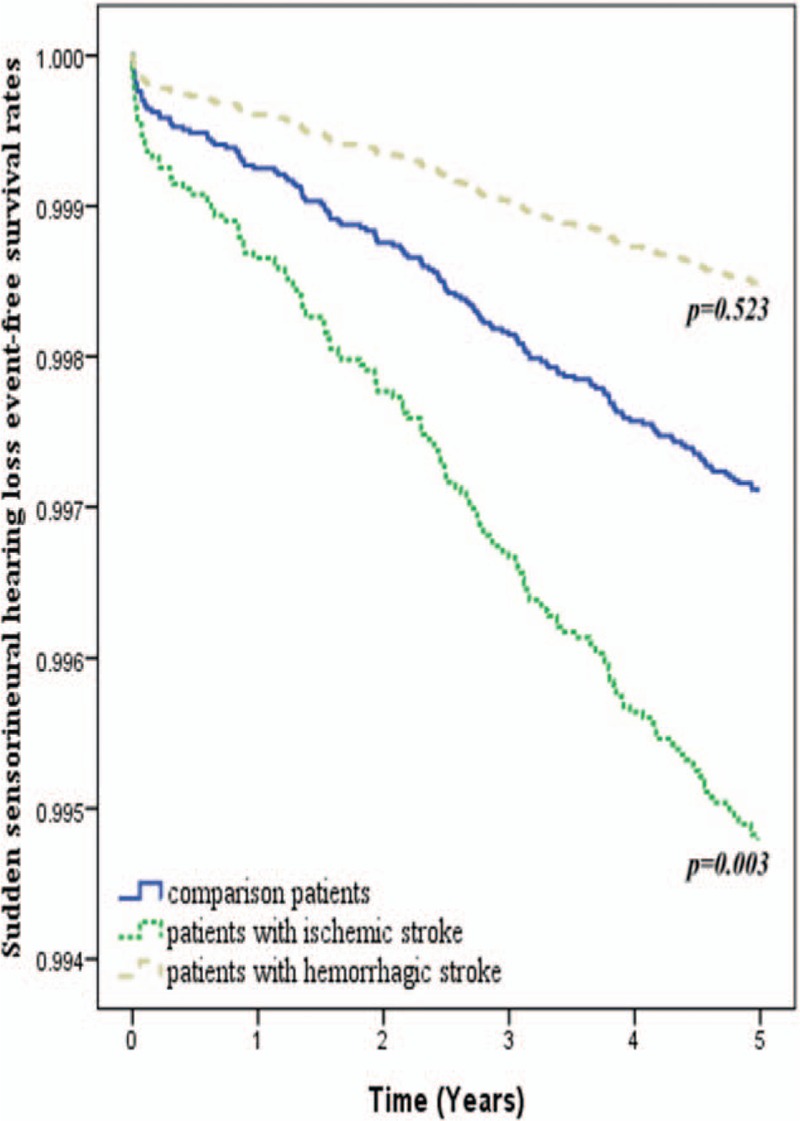
Sudden sensorineural hearing loss-free rates among patients with ischemic stroke, patients with hemorrhagic stroke, and patients without stroke during the 5-year follow-up period.

Kaplan–Meier analysis of the 5-year SSNHL-free rates in individual cohorts was implemented in conjunction with the log-rank test to examine differences in the rates between the hemorrhagic stroke group and the nonstroke group, and also between the ischemic stroke group and the nonstroke group (Fig. 2). Kaplan–Meier results conflicted with results obtained from Cox proportional-hazard regression analysis, wherein patients with ischemic stroke presented a significantly lower 5-year SSNHL-free rate than patients in the comparison cohort (log-rank test, *P* = 0.003). Patients who had undergone hemorrhagic stroke presented a higher, but nonsignificant, SSNHL-free rate than patients in the comparison cohort (log-rank test, *P* = 0.523).

### 3.5 Steroid use and the risk of SSNHL

Stroke patients who underwent steroid therapy during hospitalization presented an increased risk of SSNHL that was more than 5 times greater than that of patients without stroke (adjusted HR 5.14, 95% CI 2.08–12.75, *P* < 0.001). Conversely, stroke patients who did not undergo steroid therapy during hospitalization presented a lower risk of SSNHL (adjusted HR 0.31, 95% CI 0.11–0.86, *P* < 0.05) than did nonstroke patients (Table 3).

### 3.6 Time-independent hazard of poststroke SSNHL

To determine whether stroke is a time-dependent risk factor for SSNHL, patients were divided into 3 subgroups according to the duration of follow-up: 1, 3, or 5 years (Table 3). In all 3 follow-up periods, the risk of developing SSNHL in the stroke group was significantly higher than in the comparison group, both before and after adjustment for potential confounders (all *P* ≤ 0.001). Notably, the highest covariate-adjusted HR was observed in stroke patients who were followed up for 1 year (HR 5.65, 95% CI 3.07–10.41, *P* < 0.001).

### 3.7 Age-specific and nonsex-specific hazard of poststroke SSNHL

We investigated whether stroke is an age or sex-dependent (Table 3) risk factor for SSNHL. After adjusting for potential confounders, our results revealed that the risk of developing SSNHL faced by stroke patients aged 40 to 59 years is 2.39 times higher than that of nonstroke subjects in the same age group (95% CI 1.40–4.09, *P* < 0.01). Stroke patients of either sexes presented a higher covariate-adjusted risk of developing SSNHL than did nonstroke patients of the corresponding sex (both *P* < 0.05); however, the HR of male stroke patients was higher than that of female stroke patients (adjusted HRs 1.78 and 1.67, respectively).

## 4 Discussion

### 4.1 Possible explanations for the underestimation of poststroke SSNHL

In 2010, the worldwide prevalence of stroke was 33 million, and 16.9 million of these were first-time stroke patients.^[14]^ Owing to significant advances in emergency medicine and acute stroke care, approximately two-thirds of patients survive their stroke; however, half of them are left disabled and dependent.^[15]^ Poststroke disability has a profound impact on patients and their families and also imposes a significant burden on society and healthcare expenditures.^[16]^ It has been estimated that 25% to 74% of stroke survivors experience significant functional disabilities in mobility, the activities of daily living, social integration, and gainful employment, thereby necessitating the assistance of caregivers.^[17,18]^ Several clinical guidelines have been established to summarize evidence-based recommendations for the interdisciplinary management of stroke survivors and caregivers.^[18]^ However, these guidelines call for greater treatment emphasis on motor impairments and cognitive abilities, such as strategies to improve short-term memory, language comprehension, orientation, safety awareness, and judgment.^[17]^ Recommendations pertaining to the identification, assessment, and rehabilitation of hearing deficits in stroke patients remain unsophisticated and somewhat limited.^[17,18]^

Although the exact incidence of SSNHL in stroke patients is unknown,^[19]^ we found that the incidence of SSNHL in stroke patients was low (1.19 per 1000 patient-years). However, hearing loss has been identified in approximately 60% to 80% of stroke sufferers,^[4,20,21]^ and Edwards et al^[17]^ further revealed that as many as 86% of hearing loss cases remain undetected in patients assessed within 10 days of stroke onset. These findings suggest that poststroke SSNHL is crucial, but has been clinically underestimated.

Several factors may explain the underestimation of poststoke SSNHL. First, poststroke hearing impairments are typically less obvious than other symptoms, such as dysphasia, loss of motor function, or visual deficits. Thus, hearing impairment has not been investigated as extensively as other stroke-related outcomes.^[4]^ Second, hearing impairment in stroke patients has historically been regarded as inevitable—a functional decline that occurs naturally with age. Hence, very little effort has gone towards treating this neurological deficit. Indeed, most stroke patients are so concerned with recovering from major stroke-related sequelae that they are barely aware of a loss in auditory function. It is unlikely that clinicians would consider referring patients for auditory assessment in the absence of observed indicators.

Furthermore, administering hearing tests on stroke patients in a soundproof room is a risky procedure, especially for those in critical condition or those suffering from cognitive or functional impairments. It is not possible to identify SSNHL in unconscious stroke patients. For patients who are stable enough to undergo hearing assessment, the time required to identify poststroke hearing loss is likely to exceed 72 hours, which is greater than the maximum duration required to diagnose SSNHL.^[1]^

### 4.2 Clinical implications

Our study carries meaningful diagnostic implications. Using epidemiological evidence from a large-scale registry of 76,020 patients in a 5-year cohort design, we succeeded in identifying a prospective link between the diagnosis of stroke and the subsequent development of SSNHL. After adjusting for co-variables, the risk of developing SSNHL was higher for stroke patients than for individuals without stroke. Specifically, over a follow-up period of 1 year, stroke patients were 5.65 times more likely to develop SSNHL than were patients without stroke. Furthermore, the actual number of SSNHL cases in the stroke group may have been underestimated due to the fact that SSNHL may have gone undiagnosed in patients who were bed-ridden or who suffered from cognitive impairment. Given the strong likelihood of stroke as a predictor for the development of SSNHL, early recognition of SSNHL is indicated as part of a broader cerebrovascular event. We suggest that clinicians carefully investigate the possibility of subsequent hearing impairments among stroke patients.

Hearing loss may undermine a patient's ability to communicate, which can be highly stressful for the patient and lead to rehabilitation nonadherence. A failure to account for poststroke hearing loss could also cause patients to become socially withdrawn. Thus, it is important to raise awareness among clinicians regarding the need for management strategies to treat poststroke hearing loss. Close monitoring of hearing status among stroke patients may facilitate early detection and timely management of comorbid hearing deficits through the fitting of hearing aids. Enhancing one's ability to communicate can help to relieve their psychological burden and facilitate rehabilitation.

### 4.3 Impact of stroke subtypes on SSNHL risk

In this study, patients with ischemic stroke faced an 80% increase in the risk of developing SSNHL during a 5-year follow-up period, compared with nonstroke patients. Hemorrhagic stroke was not significantly associated with an increase in the risk of SSNHL. Theoretically, both types of stroke could affect the auditory pathway.^[4]^ Previous case reports of central hemorrhage have also documented auditory dysfunction in patients.^[22–24]^ Further research will be required to clarify our findings and to explain how stroke subtypes affect the risk of SSNHL.

### 4.4 Steroids and the risk of SSNHL

To date, steroid therapy is the most common “standard” treatment option for idiopathic SSNHL.^[1]^ However, no previous study has examined the role of steroids in poststroke SSNHL.^[1,25]^ Using a nationwide database to evaluate a high volume of patients, we observed that stroke patients who underwent steroid therapy were 5.14 times more likely than nonstroke patients to develop SSNHL. Conversely, stroke patients who did not undergo steroid therapy presented a lower risk of developing SSNHL, compared with nonstroke patients. To our knowledge, this is the first evidence-based study to examine the potential effect of steroids on poststroke SSNHL.^[1]^

Previous therapeutic observations of idiopathic SSNHL suggested that steroids may be beneficial for stroke patients at risk of SSNHL, with researchers positing that steroids can improve cochlear blood flow by reducing swelling secondary to ischemia.^[25–27]^ Other researchers have also found that steroids may have protective effects against cochlear ischemia.^[27,28]^ It is therefore surprising that the epidemiologic findings of this study seem to contradict these expectations.

Nevertheless, the correlation observed between SSNHL and stroke patients using steroids cannot be interpreted as evidence against the use of steroids in the treatment of stroke patients with SSNHL. In clinical practice, there is widespread agreement regarding the use of steroids in stroke cases where vasculitis is suspected or proven.^[29]^ Thus, it could be reasoned that patients who underwent steroid therapy were subject to vascular damage of greater severity than those who did not receive steroids. If this is the case, it is reasonable to expect that stroke patients who underwent steroid therapy would face a higher risk of SSNHL of vascular origin.

### 4.5 Strengths and limitations of the study

The primary strength of this study lies in the application of a large-scale population-based survey. This approach has several advantages, such as a large sample size, minimal selection bias, and the follow-up of all cohort members (due to the fact that the NHI program is a compulsory universal healthcare system). Under the NHI, very low copayment requirements (3–15 US dollars) contribute to the high utilization of medical services, thereby reducing the possibility that the number of cases was underestimated.

However, we are somewhat guarded in our conclusions due to the limitations of this study. First, epidemiological associations may result from a sequential comorbidity between stroke and SSNHL, which does not necessarily imply biological causation. Second, Cox regression analysis was used to adjust for confounders; however, not all of the confounding factors were necessarily included in the model. For instance, the severity and location of stroke may be relevant factors underlying the association between stroke and subsequent SSNHL. Ideally, these would be included in an analysis of co-variables. Unfortunately, the ICD-9-CM coding system lacks specific diagnostic coding groups by which to differentiate the severity or location of stroke. Excluding these variables may have confounded our estimation pertaining to the degree of correlation between stroke and SSNHL.

Notably, a recent systematic review and meta-analysis, which investigated the risk factors for adult SSNHL, indicated that developing SSNHL involves a complex pathogenetic process with multiple contributing factors.^[30]^ Both acquired and inherited cardiovascular risk factors were shown to be positively associated with SSNHL. For example, heavy smoking and alcohol consumption seem to be potential risk factors for SSNHL; and genetic mutations associated with greater risk of thromboembolic events also appear to increase the risk of SSNHL. Each factor is associated with a circumstance-specific pathogenetic mechanism. Considering all potential pathogenetic factors could help identify the true mechanisms involved in the development of SSNHL after stroke. The current study obtained secondary data from the NHI Research Database; however, this does not include data from original medical records. Such data are necessary to conduct a comprehensive investigation of all potential risk factors. Therefore, further comparative longitudinal prospective studies are required to confirm a link between acquired and inherited cardiovascular risk factors and poststroke SSNHL.

Although the findings of this study can be generalized to the Taiwanese population, applicability to other ethnicities should be revalidated. Finally, the results of this population-based study require further confirmation in comparative longitudinal prospective studies.

## 5 Conclusion

The study identified a prospective link between stroke and SSNHL, which could serve as an early warning for hearing loss in stroke patients that may otherwise go unrecognized. Particularly, within a 1-year follow-up period, we observed a remarkably higher risk of SSNHL in all stroke patients (5.65-fold compared with nonstroke patients), and also for stroke patients who underwent steroid therapy during hospitalization (5.14-fold compared with nonstroke patients). This indicates that stroke patients should be treated with the utmost caution with regard to SSNHL.
