# Risk of sudden sensorineural hearing loss in stroke patients: A 5-year nationwide investigation of 44,460 patients: Erratum

**DOI:** 10.1097/MD.0000000000006217

**Published:** 2017-02-17

In the article “Risk of sudden sensorineural hearing loss in stroke patients: A 5-year nationwide investigation of 44,460 patients”,^[1]^ which appeared in Volume 95, Issue 36 of *Medicine*, the following statement should have been included:

This study was sponsored by grants from Ministry of Science and Technology (MOST 105-2314-B-010-007-MY3, NSC 102-2628-B-010-008-MY3, NSC 101-2314-B-010-068-MY3, and V105C-154), Taoyuan Armed Forces General Hospital (No.10507), The Brain Research Center (103U-001, 103AC-B16, and 103AC-B7), Taipei Veterans General Hospital (V103C-204, V104C-068, V104E9-004, V103C-062, V103E9-003, D5134C00001, 2014-02-005AC, 2015-05-010CC, and V104C-179), and Ministry of Education.
